# Web tools to perform long non-coding RNAs analysis in oncology research

**DOI:** 10.1093/database/baab047

**Published:** 2021-07-23

**Authors:** Shixing Gu, Guangjie Zhang, Qin Si, Jiawen Dai, Zhen Song, Yingshuang Wang

**Affiliations:** College of Medical Technology, Chengdu University of Traditional Chinese Medicine, No.1166 Liutai Road, Chengdu, Sichuan 611137, China; College of Medical Technology, Chengdu University of Traditional Chinese Medicine, No.1166 Liutai Road, Chengdu, Sichuan 611137, China; Department of Clinical Laboratory, Chengdu Fifth People’s Hospital, No.33 Mashi Street, Chengdu, Sichuan 611130, China; College of Medical Technology, Chengdu University of Traditional Chinese Medicine, No.1166 Liutai Road, Chengdu, Sichuan 611137, China; College of Medical Technology, Chengdu University of Traditional Chinese Medicine, No.1166 Liutai Road, Chengdu, Sichuan 611137, China; College of Medical Technology, Chengdu University of Traditional Chinese Medicine, No.1166 Liutai Road, Chengdu, Sichuan 611137, China; College of Medical Technology, Chengdu University of Traditional Chinese Medicine, No.1166 Liutai Road, Chengdu, Sichuan 611137, China

## Abstract

Accumulated evidence suggests that the widely expressed long-non-coding RNAs (lncRNAs) are involved in biogenesis. Some aberrant lncRNAs are closely related to pathological changes, for instance, in cancer. Both in tumorigenesis and cancer progression, depending on the interplay with cellular molecules, lncRNAs can modulate transcriptional interference, chromatin remodeling, post-translational regulation and protein modification, and further interfere with signaling pathways. Aiming to the diagnosis/ prognosis markers or potential therapeutical targets, it is important to figure out the specific mechanism and the tissue-specific expressing patterns of lncRNAs. Generally, the bioinformatics analysis is the first step. More and more *in silico* databases are increasing. But the existing integrative online platforms’ functions are not only having their unique features but also share some common features, which may lead to a waste of time for researchers. Here, we reviewed these web tools according to the functions. For each database, we clarified the data source, analysis method and the evidence that the analysis result is derived from. This review also illustrated examples in practical use for a specific lncRNA by these web tools. It will provide convenience for researchers to quickly choose the appropriate bioinformatics web tools in oncology studies.

## Introduction

Long-non-coding RNAs (lncRNAs) are a heterogeneous category of non-coding RNAs containing microRNA, snoRNA, rRNA, tRNA and so on, and which are defined as RNAs longer than 200 nucleotides lacking coding potential (1, 2).

Emerging studies continuously revealed lncRNAs involving in the biological processes (3, 4). By interacting with specific gene sequences, targeting mRNAs or proteins, lncRNAs work as signal, decoy, scaffold or guide and function in transcriptional interference, chromatin remodeling, post-translational regulation and protein modification (5). Deregulated lncRNAs participate in the occurrence and development of diseases, especially in tumors. The important roles of lncRNAs have been verified in tumorigenesis, such as genome instability (6–8), promoting proliferation (9), evading growth suppressors (10), resisting apoptosis (11) and angiogenesis (12). In tumor progression, lncRNAs have also been found to regulate several steps of metastasis (13–15), such as cell invasion, migration and avoidance of immune destruction (16). Some lncRNAs are tissue- or cancer-type-specific expression, which may contribute to the diagnosis (17), survival prediction (18) and provide potential therapy targets (19).

**Figure 1.**
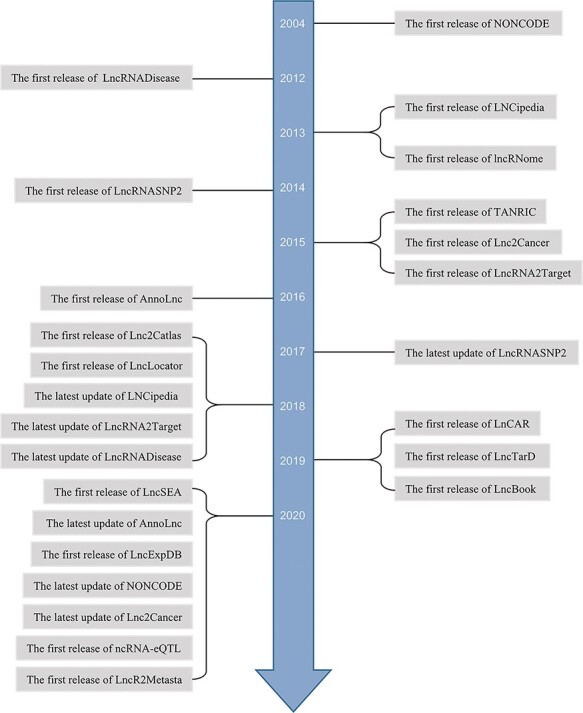
Timeline of lncRNA-related databases.

Some lncRNAs are screened by high-throughput sequencing (20, 21). In general, to investigate the roles and mechanisms of lncRNAs, studies estimate the expression differences and predicate localization and interaction targets. To analyze clinical relevance, survival analysis and prognostic assessment have been applied to specific cancer. Afterwards, *in vitro* and *in vivo* experiments are designed to perform validation. In most of these studies, bioinformatics analysis is the first step. With the contribution of increasing high-throughput sequencing data and the opening source of clinical information, more and more *in silico* databases are arising. Meanwhile, manually curated databases of experimental lncRNAs are also emerging (Figure 1). Some webtools for lncRNAs analyses are multifunctional, interactive and user-friendly and less time-consuming especially for researchers who lack programming capability. They can get novel or previously unannotated lncRNAs to guide their further studies.

However, existing integrative online platforms’ functions are partially overlapped and have their own features, which make it difficult for users to choose. Here, we reviewed these web tools according to the functions and clearly displayed the functions by tables, including both basic information (Table 1) and special features (Table 2), providing convenience for researchers to quickly choose the right one for their further studies.

**Table 1.** Basic information of reviewed LncRNA analysis web tools

	Contents Name	Data sources	Quoted rate (Until 2021)	URL	DOI	Country	The first release time The update time
General platforms	lncRNome	Gencode Release, Ensembl, HGNC, NCBI	88	http://genome.igib.res.in/lncRNome	10.1093/database/bat034	India	2013
	LNCipedia	LncRNAdb, Broad Institute, Ensembl, Gencode, Refseq, NONCODE, FANTOM	402	https://lncipedia.org	10.1093/nar/gks915 10.1093/nar/gky1031	Belgium	2013 2018
	NONCODE	Ensembl, RefSeq, lncRNAdb, Incipedia, GenBank	1006	http://www.noncode.org/	10.1093/nar/gki041 10.1093/nar/gkx1107	China	2004 2015 2018 2020
	LncBook	GENCODE, NONCODE, LNCipedia, MiTranscriptome beta	39	http://bigd.big.ac.cn/lncbook	10.1093/nar/gky960	China	2019
	AnnoLnc	CGHub, GENCONE, LncRNAdb, UCSC, GO, GEO	28	http://annolnc.gao-lab.org/	10.1186/s12864-016-3287-9 10.1093/nar/gkaa368	China	2016 2020
	LncExpDB	GENCODE, NONCODE, LNCipedia, RefLnc, LncBook, FANTOM-CAT	2	https://bigd.big.ac.cn/lncexpdb	10.1093/nar/gkaa850	China	2020
	LncSEA	Cistrome, NCBI, TCGA, ENCODE, LncMap, Lnc2Cancer2.0, LncRNADisease 2.0, GTRD	1	http://bio.liclab.net/LncSEA/index.php	10.1093/nar/gkaa806	China	2020
Specific function analysis	LncRNASNP2	TCGA, COSMIC	57	http://bioinfo.life.hust.edu.cn/lncRNASNP#!/	10.1093/nar/gku1000 10.1093/nar/gkx1004	China	2014 2017
	LncRNA2Target	Pubmed, GEO	198	http://123.59.132.21/lncrna2target/index.jsp	10.1093/nar/gku1173 10.1093/nar/gky1051	China	2015 2018
	LncLocator	RNALocate	79	http://www.csbio.sjtu.edu.cn/bioinf/lncLocator/	10.1093/bioinformatics/bty085	China, Netherlands	2018
	LncTarD	TCGA, Pubmed	9	http://bio-bigdata.hrbmu.edu.cn/LncTarD/index.jsp	10.1093/nar/gkz985	China	2019
Clinical significance analysis and prediction	LncRNADisease	Pubmed	624	http://cmbi.bjmu.edu.cn/lncrnadisease	10.1093/nar/gks1099 10.1093/nar/gky905	China	2012 2018
	TANRIC	CouchDB	274	https://ibl.mdanderson.org/tanric/_design/basic/main.html	10.1158/0008-5472.Can-15-0273	United States	2015
	Lnc2Cancer	Pubmed, TCGA, GEO, Ensembl, RefSeq, NONCODE, COSMIC, OMIM	226	http://www.bio-bigdata.net/lnc2cancer	10.1093/nar/gkv1094 10.1093/nar/gkaa1006	China	2015 2020
	Lnc2Catlas	GENCODE, TCGA, dbSNP, MalaCards, DisGeNET, Pubmed	11	https://lnc2catlas.bioinfotech.org/home/	10.1038/s41598-018-20232-4	China	2018
	LnCAR	Affymetrix, Aglient, Illumina	8	https://lncar.renlab.org/	10.1158/0008-5472.Can-18-2169	China	2019
	LncR2Metasta	Pubmed, Enterz, Ensembl	N/A	http://lncr2metasta.wchoda.com/search	10.1093/bib/bbaa178	China, Netherlands	2020
	ncRNA-eQTL	TCGA, GWAS, GENCODE	6	http://ibi.hzau.edu.cn/ncRNA-eQTL/	10.1093/nar/gkz711	China	2020

**Table 2.** Function characteristics of LncRNA analysis web tools

	*Sequence information*								*Molecular function*					*Clinical relevance*							
Name	Sequence analysis	Transcript information	Conservative	Secondary structure	Methylation	SNP	Coding potential	Subcell location	Gene interaction	mRNA interaction	miRNA interaction	Protein interaction	Signal pathway	Expression difference	Expression profiles	Variation	Survival analysis	Cancer stage	Metastasis	Disease-related lncRNA	Drug
lncRNome	√	√	√	√	√	√	√				√	√				√				√	
LNCipedia	√	√	√	√			√				√										
NONCODE	√	√	√	√	√	√	√								√	√				√	
LncBook	√	√			√	√	√				√			√		√				√	
AnnoLnc	√	√	√	√		√	√	√			√	√		√	√	√				√	
LncExpDB	√	√					√	√		√				√	√					√	
LncSEA	√	√	√		√	√	√	√		√	√	√		√	√		√	√	√	√	√
LncRNASNP	√	√	√			√					√			√	√	√				√	
LncRNA2Target	√	√		√					√					√	√		√			√	
LncLocator	√							√													
LncTarD	√	√			√					√		√		√		√			√	√	√
LncRNADisease	√	√						√	√	√	√	√								√	
TANRIC	√	√							√	√	√	√	√	√	√	√	√	√		√	√
Lnc2Cancer	√	√			√		√	√		√	√			√		√	√	√	√	√	√
Lnc2Catlas	√	√		√		√						√	√	√						√	
LnCAR	√	√	√	√			√						√	√	√	√	√	√	√	√	√
LncR2Metasta	√	√											√		√				√	√	
ncRNA-eQTL	√	√				√					√			√		√	√	√	√	√	

## General platforms

### lncRNome

lncRNome is a database released in 2013 by CSIR (Centre of Scientific and Industrial Research)-Institute of India genomics and Integrative Biology (22). Database statistics show 10 840 of lncRNA genes and 17 547 alternative splice variants. It contains the following sub-class datasets: 4491 RNA–protein interactions, 310 010 variations mapping to lncRNAs and more than 40 000 of epigenetic markers mapping around lncRNA TSS (transcription start site).

In terms of data reference and processing, some of the data in the ‘General’ module come from Gencode release 12 (23). Data such as Disease Association and PMID are derived from manually collated literature. LncRNA sequence and single nucleotide polymorphisms (SNPs) from UCSC (University of California, Santa Cruz) Genome Browser Database (24). RNA structures are calculated by using RNAfold and protein–RNA interactions that are partly based on experimental data sets and a computational prediction method for predicting the residues in RNA involving the support vector machine.

lncRNome provides (i) sequence information: including sequence analysis, basic transcript information, conservative, secondary structure, methylation, SNP, protein-coding potential and expression, (ii) molecular function: including lncRNA–miRNA interaction and lncRNA–protein interaction based on experimental data sets and calculations and (iii) clinical relevance: including variation and disease-related lncRNA.

In the ‘General’ module, researchers can query the expression levels of specific lncRNA in different cancer types and its correlation with other diseases. lncRNome is helpful in large-scale screening experiments, such as expression analysis of disease-associated lncRNAs. Claudia Cava *et al.* obtained 10 167 lncRNAs reported from lncRNome when they set up co-expression networks among miRNAs, lncRNAs and mRNAs in normal tissues (25). Kaili Luo *et al.* analyzed data from lncRNome along with the Atlas of Non-coding RNAs in Cancer (TANRIC) to identify novel lncRNAs potentially associated with colorectal cancer (CRC). LncRNA CASC9 was picked for further in-depth study, which was highly expressed and related to poor survival in colon cancer. It showed that CASA9 is a promising prognostic predictor and potential therapeutic target (26).

### LNCipedia

LNCipedia (27) was first released in 2012 by the Center for Medical Genetics, Ghent University, Belgium, and then, it has been updated to LNCipedia 5 (28) in 2018. The total number of genes in the database is 56 946 (127 802 transcripts), and the high confidence set contains 49 372 genes (107 039 transcripts).

LncRNA data come from the following databases: LncRNAdb (29), Broad Institute (Human Body Map lincRNAs), Ensembl (30), GENCODE (31), etc. The developers used the RNAfold algorithm to generate the secondary structure and point diagrams with pairing probabilities and applied MirTarget2 (32) algorithm to predict miRNA seeds. The protein-coding potential is evaluated by using two algorithms, Coding-Potential Calculator and PRIDE database at EMBL-EBI (33).

LNCipedia provides (i) sequence information, including expression, sequence analysis, basic transcript information, conservative, secondary structure and protein-coding potential and (ii) molecular function: including miRNA seed prediction.

LNCipedia is a comprehensive database with multiple functions. Alberto Cedro-Tanda *et al.* annotated the data of HT-seq raw counts with LNCipedia to get lncRNAs and filter the low expressed row count data in their study of exploring lncRNA landscape in breast cancer. Data were further handled to portray lncRNAs in different subtypes of breast cancer. Subsequently, they chose lncRNA AC009283.1 to explore the functions in Human Epidermal Growth Factor Receptor 2-enriched breast cancer. Results showed that AC009283.1 regulates genes involving multiple biological progresses (34).

### NONCODE

NONCODE was first released by the Chinese Academy of Sciences in 2004 (35) and the database has been upgraded several times by 2020, with the latest update, NONCODEV6 (14), released in November 2020. NONCODEV6 contains 644 510 lncRNA transcripts in 39 species, collecting 16 animals and 23 plants, with humans containing 173 112 lncRNA transcripts.

As for data collection, NONCODEv6-related lncRNA data are collected from the following databases: Ensembl (36), RefSeq (37), LncRNAdb (38), LNCipedia (28), CANTATAdb (39) and older versions of NONCODE. The developers collected 13 749 lncRNA records and disease-related information through documentation and mutation analysis of genome-wide association study (GWAS) data (40). NONCODEv6 summarizes lncRNA cancer-related information from six related databases [including: LncSpA (41), Lnc2Cancer (42), LncRNAWiki (43), LncTarD (44), MNDR (45) and LncRNADisease (46)] and the literature.

NONCODE provides (i) sequence information: including sequence analysis, basic transcript information, conservative, secondary structure, methylation, SNP, protein-coding potential and expression and (ii) clinical relevance: including variation, cancer cell lines expression profiles and disease-related lncRNAs.

NONCODE provides a user-friendly interface, supporting the collation of lncRNA-related diseases by collecting six human exosome data sets (six tumor cell lines and four tissues) from (The Gene Expression Omnibus) GEO (47) to support the collation of lncRNA-related diseases. In addition, users can query information about the SNP disease of interesting lncRNAs and view the expression spectrum of lncRNA in human tissue provided by the database.

Ching-Ching Yeh *et al.* explored the molecular mechanism of lncRNA *NDRG1-OT1* on *NDRG1*. And they constructed six fragments according to the 2D structure of *NDRG1-OT1* predicted by NONCODE. Furthermore, the results of luciferase assays with different fragments indicated that different fragments of *NDRG1-OT1* had different impacts on the same target gene, *NDRG1*, in breast cancer cells under hypoxia (48).

### LncBook

LncBook is a comprehensive lncRNA online analytics platform released by the Chinese Academy of Sciences in 2018 (49). It integrates multi-omics data from databases such as GENCODE v27 (31), NONCODE v5 (50), LNCipedia v4.1 (51), Mi-Transcriptome beta (1) and LncRNAWiki (43) in a structured manner and obtains 27 044 non-redundant lncRNA transcripts which belong to 140 362 gene sites.

To perform functional analysis, LncBook uses three algorithms to predict lncRNA-coding potential: (Laboratory of the Government Chemist) LGC, (53) Coding-Potential Assessment Tool (CPAT) (52) and PLEK (predictor of long non-coding RNAs and messenger RNAs based on an improved k-mer scheme) (53); TargetScan (54) and miRanda (55) to predict the interactions between lncRNA and miRNA; identify new lncRNAs with Human Protein Atlas (56), FastaQC, Trimmomatic (57), Hisat2 (58), StringTie (55) and CuffCompare (59). All the analyses can be easily applied by browsing or lncRNA ID/sequence searching.

LncBook provides (i) sequence information, including, sequence analysis, basic transcript information, methylation, SNP, expression and protein-coding potential prediction, (ii) molecular function, including interactions between lncRNA and miRNA and (iii) clinical relevance, including differential expression between cancer and normal tissues, genomic variation and disease-related lncRNAs.

In the ‘Diseases’ module, the platform describes each lncRNA with symbol, dysfunction type, detailed description, disease name, MeSH Ontology and PMID. LncBook includes 3772 experimentally supported disease-associated lncRNAs, and 9798 lncRNAs may be associated with diseases through methylation, genomic variation and lncRNA–miRNA interaction predictions (49). For each lncRNA, LncBook provides methylation levels for the promoter and body region ofnormal tissues and cancer samples of nine cancers, and these things are summarized in tables and visualized expression difference in a chart. In LncBook’s cancer prediction mechanism, the algorithm suggests that a lncRNA is associated with cancer if high or low methylation persists in at least eight types of cancer.

In practice, to research the molecular mechanism of LINC00673 in breast cancer, Kun Qiao *et al.* used Lncbook to explore potential miRNAs that may be targeted by LINC00673 and MARK4.258 miRNAs were identified and of which 40 miRNAs that had been claimed to be associated with cancers were selected for further study. The expression of 18 miRNAs was distinctly increased along with the knockdown of LINC00673. Then, miR-515-5p was chose to verify that it could bind to LINC00673 and MARK4 in breast cancer cells (60).

### AnnoLnc2

AnnoLnc was first released by Peking University in China in 2016 (61) and updated to AnnoLnc2 (62) in 2020. Developers collected information about lncRNA expression, subcellular location, SNP site, etc., from databases such as CGHub (63), GENCODE, LncRNAdb v2 (38), UCSC (24), gene ontology (GO) and GEO (47). The LncRNA–protein interaction is based on experimental support and sequence prediction.

AnnoLnc2 provides (i) sequence information: including sequence analysis, basic transcript information, conservative, secondary structure, SNP, coding potential and co-expression, (ii) molecular function: including subcellular location, prediction lncRNA–miRNA interaction and lncRNA–protein interaction and (iii) clinical relevance: including variation, expression difference between cancer and normal tissues, cell line expression profiles and disease-related lncRNAs.

In general, while querying the database with lncRNA sequence in Fasta format, the detailed information about the lncRNA can be downloaded or browsed online including its structure, location, expression, interactions and so on. These data can be analyzed and applied to further studies.

AnnoLnc2 is a one-stop portal to annotate novel lncRNAs with multiple functions. The function of predicting lncRNA–miRNA interaction was enrolled in their published studies (64, 65). In the research about lncRNA SNHG6 in CRC, AnnoLnc2 was utilized to compare SNHG6 expression levels in different cancers. Data showed that the expression of SNHG6 was relatively high in CRC compared with that of many other cancers (66). Meanwhile, Mingliang Liu *et al.* used AnnoLnc2 to predict the secondary structure of lncRNA CF129, which was further validated stabilization of p53 protein in pancreatic cancer progression (67).

### LncExpDB

LncExpDB was released by China’s National Bio-Information Center in 2020 (68). The database contains the expression of the lncRNA genes from 101 293 individuals in 337 biological environments and 1977 samples under nine biological conditions. In addition, the developers identified 25 191 signature genes and obtained more than 28.44 million lncRNA–mRNA interactions.

In terms of data sources and processing, LncExpDB-integrated lncRNA transcripts from databases such as LncBook v1.2 (49), RefLnc (69), GENCODE V33 (70), CHESS v2.2 (71) and FANTOM-CAT (72). The developers used four algorithms [LGC, CPC2 (73), CPAT (52) and PLEK (53)] to make the coding potential and the co-expression network to predict lncRNA–mRNA interactions.

LncExpDB provides (i) sequence information: including sequence analysis, basic transcript information and coding potential, (ii) molecular function: including subcellular localization and lncRNA–mRNA interaction and (iii) clinical relevance: including differential expression between cancer and normal tissues, cancer cell lines expression profiles and disease-related lncRNAs.

Expression capacity refers to the differential expression of characteristic lncRNA genes in specific cell line, tissue-specific tables, exosomes, cancers or viral infections. On the interface, users can browse the expressions of interested lncRNAs in nine different biological contexts, including cancer tissue/cell lines, normal tissue/cell line, exosome, cell differentiation, subcellular localization, preimplantation embryo, organ development, virus infection and circadian rhythm. Based on a large amount of RNA-seq data, a total of 2907 cancer cells were identified, and oncologists were able to estimate the expression of the lncRNA’s biological function in cancer cells (68). In the cancer cell line option in the context module, the visual expression of interested lncRNA can be gained in cancer cell line.

### LncSEA

LncSEA is a comprehensive database of annotation and enrichment analysis of lncRNA published by Harbin Medical University in China in 2020 (74). The developers integrated lncRNA datasets from more than 20 databases, such as the genome region of the sample from Cistrome (75) and NCBI, which collected lncRNA related to cancer survival from The Cancer Genome Atlas (TCGA) (76) and information on diseases and drugs from databases such as Lnc2Cancer2.0 (77) and LncRNADisease2.0 (46). The relationship between lncRNA–mRNA and lncRNA–miRNA was downloaded from LncACTdb2.0 (78).

LncSEA provides (i) sequence information: including sequence analysis, basic transcript information, conservative, methylation, SNP, coding potential and expression, (ii) molecular function: including subcellular locations, lncRNA–mRNA interaction, lncRNA–miRNA interaction and RNA-binding protein and (iii) clinical relevance: including cancer and normal tissues expression difference, cell lines expression profiles, survival analysis, cancer clinical stage, metastasis, lncRNA-related diseases and drugs.

**Figure 2.**
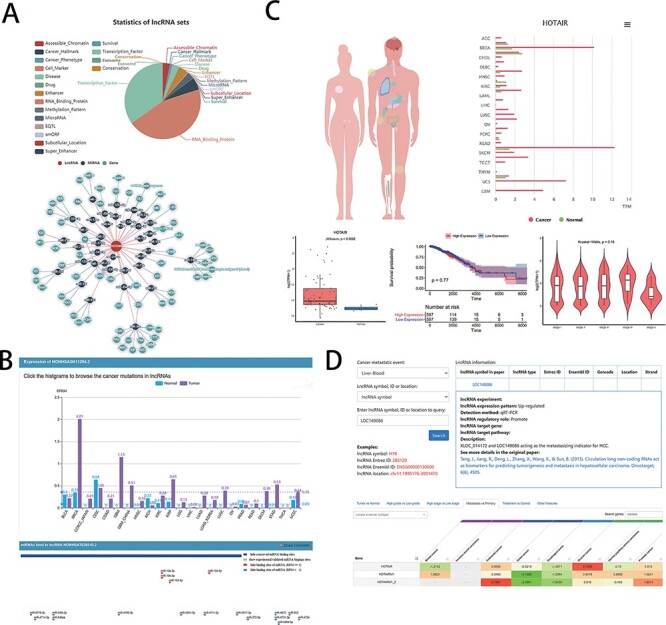
Examples of graphical analysis outputs generated for the LncRNA HOTAIR in cancers using web platforms: (A) Functions (i) and relevant ceRNA network diagram (ii) of HOTAIR was analyzed by LncSEA in 18 kinds of reference data sets, including Accessible chromatin, Cancer hallmark, Cancer phenotype, Cell marker, Disease, Drug, Enhancer, RNA-binding protein, Methylation pattern, MicroRNA, EQTL, smORF, Subcellular localization, Super enhancer, Survival, TF, Exosome and Conservation. (B) The expression differences of HOTAIR in 21 types of cancer and normal tissues was shown in histogram (i) by LncRNASNP2. And the scatter diagram (ii) was shown the binding sites of miRNAs predicted by LncRNASNP2. (C). Lnc2Cancer output an overview of the statistics of lncRNA HOTAIR based on the human map and expression charts of cancer tissues (i). Box plot (ii) was used to compare the expression of HOTAIR between specific cancer and normal samples. (iii) Kaplan–Meier plot showing overall survival in higher (shown in red) and lower (shown in blue) HOTAIR expression groups in of COAD patients. (iv)Violin plots showing lncRNA expressing levels among stage I, II, III and IV COAD samples. D. LncR2Metasta (i) displayed the cancer metastasis events in which lncRNA HOTAIR may involve in and provided supported information such as expression patterns, detection methods and origin research paper. LnCAR (ii) shows the degree of metastasis expression of HOTAIR in different cancers by heat map, where green transition red represents the transition from low expression to high expression.

The developers provided clinical research assistance to users in the LncSEA (Figure 2A), such as disease (each disease collection contains a list of lncRNA associated with the disease), drug (containing two subsets of experimental support and algorithmic prediction), cancer hallmark (including metastasis, apoptosis, prognosis, etc.) and cancer phenotype (database including 40 cancers and 325 phenotypes). In addition, LncSEA provides feature annotations and enrichment analysis capabilities. Oncologists can analyze the differential expression genes of lncRNA in cancers by LncSEA and use the results to study the relationship between lncRNA and cancer or drugs in preparation for the development of new target drugs. On the user interface, users can first explore a certain specific lncRNA and, second, they can click ‘drug’ in subcategory, and then, the drugs on the screen are related to the lncRNA. Third, clicking one of the drugs that have been searched for can link to other lncRNAs even diseases that are related to the drug.

LncSEA is a newly published comprehensive platform providing various lncRNA sets with annotation and enrichment analysis features by submitting lncRNA lists. The lncRNA set enrichment analyses are related to upstream regulators and downstream targets of RNAs. Visual bar and bubble chart of results can be gained friendly.

## Specific function analysis

### lncRNASNP2

LncRNASNP was first published by China Huazhong University of Science and Technology in 2014 (79) and updated to LncRNASNP2 in 2017 (80). The database provides a web tool that helps users predict the impact of SNPs on the secondary structure of lncRNA. 859 534 CosmicNCVs (Cosmic Non-Coding Variants) were collected from the COSMIC (Catalogue Of Somatic Mutations In Cancer) (81). 71 410 pathogenic variants were found on the lncRNA transcript. Cancer mutations were collected -from the TCGA (82) database and 315 234 mutated lncRNA transcripts were found in 34 cancer types. Developers used algorithms to calculate and collected experimentally supporting data to predict lncRNA-related diseases.

LncRNASNP2 provides (i) sequence information: including sequence analysis, conservative, secondary structure, basic transcript information, SNP and expression, (ii) molecular function: including miRNA–lncRNA interaction prediction and (iii) clinical relevance: including expression difference in different cancers, expression profiles in cancer cell lines, variation and disease-related lncRNAs.

For users to study disease-related information, the ‘expression’ section not only summarizes the expression of lncRNA under different tumors and mutations but also compares the expression specificity of lncRNA in different situations [Figure 2B (i)]. Basic information of miRNA provides the level of expression of each human miRNA in 28 cancers [Figure 2B (ii)]; thus, oncologists can view the classification of lncRNA-related diseases [experimental support or based on TAM (83) (The tool for annotations of human miRNAs) prediction].

LncRNASNP2 aims to provide comprehensive information about lncRNA SNPs and their effects on the lncRNA structure and function, particularly miRNA–lncRNA interaction. Wenyuan Zhao *et al.* used LncRNASNP2 to predict the targeted miRNAs of LINC02310 to further construct a regulatory network in lung adenocarcinoma (84). The potential effects of SNPs on the generation or destruction of the miRNA binding site were analyzed using lncRNASNP2 (85, 86). Different SNPs in the same allele allowed different interactions with miRNA, as further studies verified the discrepancy, such as luciferase reporter assay. At last, the study suggested A allele of SNP rs140618127 in lncRNALOC146880 may have a protective effect on non-small cell lung cancer (NSCLC) compared with genotype [G] (86).

### LncRNA2Target

LncRNA2Target was first released by Harbin Institute of Technology in China in 2014 (87) and updated to LncRNA2Target v2.0 (88) in 2019. Until today, the database contains 152 137 lncRNA–target associations from 1047 papers and 224 data sets.

Developers retrieved literature and datasets from the Pubmed and GEO (47) databases. The target gene of lncRNA was collected from all differential expression genes after knocking down or over-expression of a certain lncRNA or inferred from low-throughput experiments (such as immunoprecipitation assays, RNA pull down assays, luciferase reporter assays RT-qPCR (Real Time Quantitative PCR) and western blot).

LncRNA2Target provides (i) sequence information including sequence analysis and basic transcript information, (ii) molecular function: expression and gene interaction and (iii) clinical relevance: including expression difference, cancer cell lines, survival analysis and disease-related lncRNAs.

In clinical studies, users can directly query the disease status and expression spectrum in cancer cell lines associated with the lncRNA target gene. When users click on the target symbol, experimental evidence supporting lncRNA–target association is displayed, including links to the original literature and partial prognostic analysis.

LncRNA2Target is chiefly aiming to provide lncRNA-to-target genes experimentally verified. In the study of identifying cancer-related lncRNAs, Zihao Liu *et al.* used LncRNA2Target to obtain target genes of lncRNA and, then, select the most common target genes corresponding to more than five lncRNAs to be the features of lncRNAs. With the cancer-related lncRNAs gained from LncRNADisease database and some methods such as Deep Belief Network applied to the study, potential lncRNAs for 16 kinds of cancers were obtained (89).

### LncLocator

Recent studies have shown that the subcellular location of lncRNA provides important information for understanding their complex biological functions. LncLocator, the first web tool for predicting lncRNA subcellular positioning, was released in 2018 by Shanghai Jiao Tong University in China (90) and updated version 2.0 in 2021 (91). Humanly managed and experimentally supported RNA-related subcellular positioning entries are downloaded from the comprehensive database RNALocate (92), and the filtered baseline dataset contains 612 lncRNAs covering five subcellular compartments.

In version 1.0, K-mer (93) and deep structure abstract features are incorporated into the model construction of this study, and a supervised oversampling method called SOS (supervised oversampling) (94) is adopted. To improve prediction performance, LncLocator uses a stacked combination strategy to combine four learning machines, including random forest with features extracted by deep neural networks (RFA), supporting vector machine with features extracted by deep neural networks (SVMA), random forest with raw k-mer features (RFR) and support vector machine with raw k-mer features (SVMR).

In the 2.0 version, the developers pre-constructed a baseline dataset of lncRNA subcellular positioning for 15 cell lines. Then they used natural language models to learn word embedding and input the embedding of these learnings into the convolutional neural network, long short-term memory and multilayer perceptron to classify different cell lines effectively.

In terms of function, LncLocator can analyze lncRNA sequence, calculate and predict five subcellular locations, including nucleus, cytoplasm, ribosome, membrane and exosome. Each item has a 0–1 rating mechanism, and the higher score indicates a greater chance of reaching the cubicle. In the previously constructed benchmark data set test, the prediction accuracy of the LncLocator database was 0.59.

The functions of lncRNAs rely on the subcellular locations. For instance, lncRNAs located in cytoplasm regulate posttranscriptional expression. Practically when researchers determine the molecular mechanism of lncRNAs, LncLocator is applied to predict the locations of lncRNAs. To analyze the biological functions of lncRNA LCAT1 in lung cancer, Juze Yang *et al.* predicted the location of lncRNA LCAT1 by LncLocator. It was suggested to be located in cytoplast, indicating LCAT1 may regulate protein expression at the posttranscriptional level. Further studies verified that LCAT1 regulate functions via miR-4715-5p (95).

### LncTarD

LncTarD was released by Harbin Medical University in 2019 (44), including 33 types of cancer from TCGA, in addition 1862 articles from PubMed.

LncTarD provides (i) sequence information: including sequence analysis, methylation, basic transcript information and expression, (ii) molecular function: including lncRNA–mRNA interaction and lncRNA–protein interaction and (iii) clinical relevance: including metastasis, variation, differential expression between different cancers and normal tissues, lncRNA-mediated drugs and disease-related lncRNAs.

Using the data in LncTarD, users can visually see the relevant lncRNA target regulations of top lncRNA, disease and drug in the ‘Browser’ module. Developers provide users with a visual website to show the regulation of lncRNA target in diseases, including the biological function of the effects from lncRNAs and related drugs. On the visual dynamic chart interface, oncologists can view box charts of differences in lncRNA in various tumors and normal tissues, as well as other disease-related scatter pages or heat maps.

LncTarD is a manually curated database supported by experiments mainly focusing on diseases relevant lncRNAs. Qinchen Lu *et al.* used the database to validate the expression of lncRNA HOXA-AS2 in hepatocellular carcinoma (HCC) in TCGA, up-regulated in both cell lines and tissues of HCC. Further studies verified that the higher expression of HOXA-AS2 was correlated with poor prognosis positively and stem cell-related functions (96).

## Clinical significance analysis and prediction

### LncRNADisease

LncRNADisease is a database of lncRNA associated with disease, released by Peking University in China in 2013 (97) and updated to LncRNADisease 2.0 in 2018 (46). LncRNADisease 2.0 contains 195 395 predicted lncRNA–disease associations, 12 207 lncRNA-mRNAs, and 2368 miRNA–lncRNA regulatory relationships. It also integrates lncRNA–disease associations supported by 10 564 experiments and circRNA–disease associations supported by 1004 experiments across four species (46).

In LncRNADisease 2.0, an option provided on the search page that allows users to filter the association of lncRNA with disease through certain experimental methods and assign a confidence score to each entry by integrating experimental and computational evidence. To further explain the functional significance of disease-related lncRNAs, a visual lncRNA–miRNA–mRNA network was constructed.

In general, LncRNADisease provides (i) sequence information: expression, sequence analysis and basic transcript information, (ii) molecular function: subcellular location, gene interaction, lncRNA–protein interaction, lncRNA–mRNA interaction and lncRNA–miRNA interaction and (iii) clinical relevance: lncRNA-related diseases.

Notably, the database provides detailed descriptions of lncRNA-related disease names, dysfunction types, detailed descriptions of lncRNA–disease associations and PMIDs of the corresponding literature. After the update, the database can provide the total associated disease number for lncRNA. To make it easier for users to obtain disease information from external sources, disease names are mapped to disease ontology (DO) (98), medical subject headings (MESH) (99) and each lncRNA–disease association entry includes disease name, category, type, define and alias. Information about the total number of disease-related lncRNAs, forecasting methods, etc., is also available for users.

To obtain more NSCLC relative lncRNAs, LncRNADisease was examined to find that CBR3-AS1, a latent functional lncRNA, is up-regulated expression in NSCLC, indicating CBR3-AS1 may play roles in the occurrence of NSCLC. Further research works were validated that CBR3-AS1 regulated the biofunctions of NSCLC cells such as proliferation, migration and invasion through affecting the dynamic process of β-catenin transport that activated the Wnt signaling pathway (100).

### TANRIC

TANRIC is a web tool for analysis and prediction of lncRNA clinical significance (101). It was released by the Department of Bioinformatics and Computational Biology in 2015, and the latest update was in 2019. More than 40 different cancer types and cell lines data in TANRIC are extracted from the following databases, including TCGA, Cancer Cell Line Encyclopedia (102) and another three independent studies (GBM (103), ccRCC and LUAD).

On data processing, TANRIC lets the lncRNA expression as Reads Per Kilobase per Million mapped reads (104) to make users quickly analyze the specified lncRNA on a web page. Using the annotated lncRNA expression data and pre-calculated correlations with clinical and genomic data, which are stored in CouchDB, TANRIC can perform correlation, expression difference and survival analysis by R and output visual results directly.

In general, TANRIC provides (i) sequence information: including sequence analysis, basic transcript information and expression, (ii) molecular function, including signal pathway, gene interaction, mRNA interaction, miRNA interaction and interactions between protein and lncRNA and (iii) clinical relevance, including variation, differential expression between cancers and normal tissues, cancer cell lines expression profiles, analyzed the patient’s survival time, tumor stage and tumor grade. It also provides genomic variation, disease-related lncRNA and evaluates the effects of lncRNA expression on drug sensitivity.

In recent studies, TANRIC has been widely used (312 cited research papers, to date) to find out specific lncRNA with significant differences between cancers and normal controls. The expression status of these lncRNAs has been analyzed by TANRIC. Lu Feng *et al.* found LINC02487 was downregulated in neck squamous cell carcinoma (105). Yuxing Zhu *et al.* discovered LINC00968 was decreased in lung adenocarcinoma (106). Based on the analysis, further studies indicated that LINC02487 might act as a tumor suppressor in oral squamous cell carcinoma and LINC00968 may play roles as a diagnostic and prognostic biomarker and therapeutic target in lung adenocarcinoma.

### Lnc2Cancer

Lnc2Cancer was first released by Harbin Medical University in 2015 (107) and latest updated to Lnc2Cancer 3.0 (42) in 2020. It aims to provide integrated resource for exploring lncRNA deregulation in various human cancers. Lnc2Cancer 3.0 records 10 303 entries associate between 2659 human lncRNAs or 743 circRNAs and 216 human cancer subtypes thorough review of more than 15 000 published papers (42).

In terms of data sets, developers manually collated literature from the PubMed database on such things as ‘lncRNA cancer’ and ‘long-non-coded RNA cancer’. Lnc2Cancer provides identifiers and links to lncRNA that exist in the Ensembl and RefSeq databases. Finally, a standardized classification scheme, the International Classification of Diseases for Oncology, the 3rd Edition (ICD-O-3), annotates each type of cancer. Lnc2Cancer also provides hyperlinks to other data information, such as lncRNAs in lncRNAdb (38) and NONCODE (14), as well as cancer in the OMIM (108) and COSMIC (109) databases. In the updated version 3.0, new cancer-related lncRNA functions are identified based on uploaded single-cell datasets and implemented by R package Seurat (version 3.1.5).

Lnc2Cancer provides (i) sequence information: including sequence analysis, basic transcript information, methylation, coding ability and expression, (ii) molecular function: including subcellular location, lncRNA–mRNA interaction and lncRNA–miRNA interaction and (iii) clinical relevance: including expression difference, variation, survival analysis, cancer clinical stage, metastasis, disease-related lncRNA and drug resistance.

Lnc2Cancer provides a user-friendly interface to query a range of cancer-related lncRNAs on the ‘RNA-seq web tool’ page. It includes General Information, Differential Expression Analysis (DEA), Box Plotting [Figure 2C (ii)], Stage Plotting, Survival Analysis [Figure 2C (iii)], Similar lncRNAs Identification, Correlation Analysis, Network Construction and Transcription Factor (TF) Motif Prediction. General Information also has a global view of interested lncRNA statistics based on a human map and lncRNA chart based on cancer type [Figure 2C (i)]. Stage Plotting, which generates a violin diagram of the expression of specific lncRNA based on the patient’s pathological stages [Figure 2C (iv)], allows the user to select one of detailed cancer stages. The Correlation Analysis feature provides expression correlation analysis for two interested lncRNAs in cancer.

Zhengwei Du *et al.* identified lncRNA-meditated transcriptional dysregulation triplets (lncTDTs) in luminal and triple-negative breast cancer basing on TF, gene, lncRNA expression profiles and TF–gene interactions verified by experiment. They collected cancer-related lncRNAs from lnc2cancer, which differentially expressed in these subtypes using t-test. The specific lncRNAs were subsequently analyzed and lncTDTs aiding development of classification and treatment of breast cancer were gained ultimately (110).

### Lnc2Catlas

Since lncRNAs are involved in multiple signaling pathways, it is essential to illustrate the molecular network interplay. Lnc2Catlas (111) was released in 2018 by the Department of Biotechnology of the Beijing Institute of Radiology in China. The database contains 247 124 pairs of lncRNA–SNP, ∼2 million lncRNA–protein interactions and 6902 co-expression clusters.

Lnc2Catlas studied the relationship between lncRNA and cancer from 33 cancer expression files in TCGA (82), annotated lncRNA transcripts from GENCODE V22, SNP in lncRNA transcripts from dbSNP and cancer-related proteins from MalaCards, DisGeNET and human protein maps. Lnc2Catlas uses three computational methods to evaluate the relationship between quantitative lncRNA and cancer, including evaluating secondary structural damage (quantitatively assessed with RNAsnp), lncRNA–protein interaction (using Global Score calculations) and co-expression networks (building weighted co-expression networks based on TCGA to WGCNA). Finally, a total of 1038 articles related to lncRNA associated with cancer are compiled to augment the experimentally supporting ability in Lnc2Catlas.

Lnc2Catlas provides (i) sequence information, including sequence analysis, basic transcript information, secondary structure, SNP-structure disruption and expression, (ii) molecular function, including lncRNA–protein interaction and signal pathway and (iii) clinical relevance, including expression difference (between different cancers) and lncRNA-related diseases.

Tao Luo *et al.* explored the co-expression network of ELMO1-AS1 in Lnc2Catlas, which 3D space showed that it is associated with various diseases and proteins (112). The result implied the significance of ELMO1-AS1 in biology. ELMO1-AS1 may be a biomarker of prognostic and therapy in HCC demonstrated by further studies (112).

Lnc2Catlas provides direct access to all 33 cancers collected with a friendly visualization page. Researchers can query in the SNP-structure disruption section that SNPs of lncRNA are associated with different cancers, and by evaluating the relationship by hitting the relationship, provide the average expression level of lncRNA in 33 cancers in the expression section, show the subgraph of lncRNA in the cancer in the co-expression network section, pull down the selection of cancers of interest in the GO and pathway analysis sections and, finally, provide experimental support literature.

### LnCAR

As an open-source of high-throughput sequencing data, lnCAR (lncRNAs from Cancer Arrays) was released in 2019 by the Sun Yat-sen University, China (113). The database lnCAR contains 732 expression spectrum array datasets covering 54 893 samples of 10 cancer types (about 52 300 cases in the sample have more than seven different sets of clinical component information). Out of 107 gene expression microarray platforms, a total of 464 502 lncRNA probes were obtained to represent 26 464 lncRNAs.

In order to unify lnCAR data standards, different algorithms are used for database resources: Affymetrix (114) data sets use RMA algorithms, and for datasets from Agilent, Illumina and other platforms, the original data are graded and normalized using LIMMA packages (115), all of which are standardized at the detection level and converted to log2.

lnCAR provides (i) sequence information: including sequence analysis, basic transcript information, conservation, secondary structure and coding potential and expression, (ii) molecular function: signal pathway and (iii) clinical relevance: including differential expression between cancer and normal tissues, cell lines expression profiles, variation, survival analysis, cancer clinical stage, metastasis, disease-related lncRNA and drug response.

Jinlin Jia *et al.* analyzed the bioinformatics data from lnCAR and uncovered that lncRNA FAM83A-AS1 was highly expressed in esophageal cell squamous carcinoma (ESCC). Involving 51 ESCC tissues versus adjacent non-tumor tissues, qRT-PCR results also indicated remarkably increased FAM83A-AS1 in ESCC. Further studies were revealed that FAM83A-AS1 acted as tumorigenesis in ESCC and distinguished ESCC with high accuracy (116).

lnCAR provides users with a great deal of information to query clinical data. The database provides a visual heat map of different expressions of results and different types of survival information under different conditions, where ‘differential expression’ includes tumor vs. normal, high grade vs. low grade, high stage vs. low stage, metastasis vs. primary [Figure 2D (ii)], treatment vs. control and other features. And users can explore the relationship between lncRNA and different survival outcomes, such as total survival and recurrence-free survival for different cancer types.

### LncR2metasta

Numerous studies have shown that metastasis is a major limiting factor affecting cancer prognosis and a huge obstacle in clinical treatment. LncR2metasta was released by Wuhan University of Science and Technology in China in 2020 (117). The database collected 1283 associations between 304 lncRNAs and 39 tumor metastasis events out of 54 tumor types.

A large amount of data indicates that lncRNA is involved in a variety of cancer metastatic events (CMEs). The database manually collates lncRNAs supported by experiments in various CMEs in existing studies, in addition, conducts literature retrieval through Pubmed for insanity transfer experiments, and other data are collected from databases such as Entrez (118) and Ensembl (119) that are finally presented through web-based databases.

LncR2metasta provides (i) sequence information: including sequence analysis, basic transcript information and expression pattern, (ii) molecular function: including target gene (or pathway) of lncRNA and (iii) clinical relevance: including cancer cell lines expression profiles, CMEs and lncRNA-related diseases.

In clinical studies, research works on lncRNA-CME can help understand the role of lncRNA in metastasis and provide reliable biomarkers for early detection of metastasis and optimal treatment. Oncologists can query lncRNA in the database they are interested in [Figure 2D (i)], or they can look for lncRNA based on events related to tumor metastasis. The database also provides different transfer-related functions of lncRNA, including ‘cancer cell invasion’, ‘cancer cell migration’ and ‘cancer cell product’. Users can also select a special CME to query related lncRNA information by lncRNA symbol, ID (Entrez ID and Ensembl ID) or location.

### ncRNA-eQTL

Assessing the effects of SNPs on ncRNA expression using data from multiple cancer types will help to understand how genetic risk all-like genes promote tumor occurrence and cancer development. Non-coding RNA-expression quantitative trait loci (ncRNA-eQTL) is a database published by Huazhong Agricultural University in China in 2019 (120). There are more than 300 samples of 12 cancer types in the database.

In terms of data sources, SNP parting results, lncRNA and miRNA expression, clinical information of samples was obtained from TCGA (82), and GWAS analysis results were obtained from the GWAS catalog. In addition, the developers linked ncRNA-eQTL to known GWAS loci and patient lifetimes, and the developers also identified thousands of GWAS-related and survival-related eQTLs.

ncRNA-eQTL provides (i) sequence information, including sequence analysis, basic transcript information, expression and SNP, (ii) molecular function: miRNA interaction and (iii) clinical relevance: including differential expression between cancer and normal tissues, variation, survival analysis, cancer clinical stage, metastasis and disease-related lncRNA.

On the clinical side, to prioritize promising ncRNA-eQTL, developers link the eQTL of the database to clinical data in TCGA patients, allowing users to query eQTL that may be relevant to the total lifetime. These survival-related eQTLs and associated ncRNAs can be applied to biomarkers for prediction and prognosis. Users can also query that lncRNA-eQTL overlaps with known disease/symptom-related bits.

Maija Suvanto *et al.* explored SNPs in lncRNA regions where genetic variants are associated with breast cancer risk. Datasets from Genotype-Tissue Expression GETx and Molecular Taxonomy of Breast Cancer International Consortium were brought into eQTL analysis and further verification by ncRNA-eQTL online. SNPs uc.184 and uc.313 were presumed associated with breast cancer risk, providing candidate loci for further study on molecular mechanisms in breast cancer (121).

## Summary and discussion

Accumulating research works gradually uncovered the biogenesis in which the widely expressed lncRNAs are involved. These processes are closely related to their subcellular localizations and specific functions. Depending on the interactions with gene, RNAs or proteins, lncRNAs can modulate transcriptional interference, chromatin remodeling, post-translational regulation and protein modification, and further interfere with the signaling pathways. Ultimately, aberrant functions step over the normal biological process to physiopathological contexts, such as carcinogenesis and progressing.

**Figure 3.**
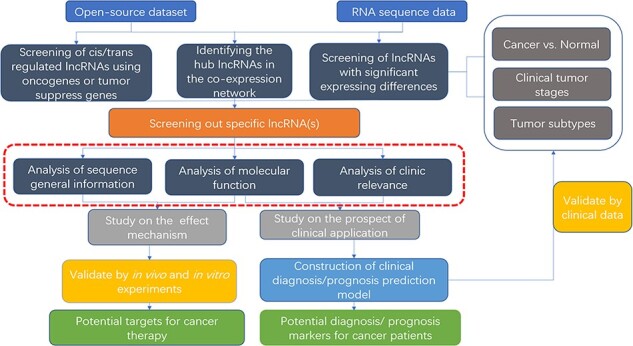
Flow chart of lncRNA in silico analysis and validation in oncology research. This flow chart indicates the general process of lncRNA analysis and validation in cancer research, the purpose of which are searching for characteristic markers of diagnosis/prognosis or potential molecular targets for treatment. Both data from open-source datasets or high-throughput sequencing results of clinical samples can be utilized. The specific lncRNA can be screened out by analysis of expressing differences, co-expressing network or cis/trans regulating of oncogenes or tumor suppressors. To clarify its roles, researchers can use web tools (the red dotted line area, which is described detailed in Table 2) to analysis the specific functions, including sequence general information, molecular function, and clinic relevance. It is essential to verify the results of the functional analysis by molecular experiments and animal models. For the establishment of diagnostic/prognostic markers, in addition to validation, lncRNAs also need to be compared with the existing clinical criteria to evaluation.

To help unravel the mechanisms of lncRNAs functions in cancers, bioinformatics databases to analyze lncRNAs are arising. To study the roles and mechanisms of lncRNAs in relevant tumors, oncologists first screen for specific lncRNAs. Some databases based on computer language provide visualized tumor-related lncRNA tables or heat maps, which can help obtain the target lncRNA. When verifying the function of the target lncRNA for further study, some databases provide co-expression nets such as interacting proteins, mRNAs and miRNAs. And in clinical correlation analysis, some databases provide prognosis analysis and disease correlation analysis for visualization. On these grounds, they can design in *vivo/in vitro* experiments or expand the clinical sample size for verification (Figure 3). Unlike previous databases that require users to know programming language and download plenty of data resources to analyze, some newly established web tools are more convenient and efficient for users. Although there are some good reviews, the databases included are relatively early (122) or minor attention to disease-related databases (123).

We also illustrated examples of graphical analysis outputs generated for the lncRNA HOTAIR in cancers using web platforms, hoping that worldwide oncology researchers can use these web tools in exploring the unannotated lncRNAs or known lncRNA with novel functions. These tissue-specific or condition-specific expressing patterns of lncRNAs suggest that they are potential biomarkers in clinical diagnosis and prognosis analysis, meaning these specific roles that they played in the cancer cell fate provide a rationale to target them in potential therapy.
